# The association between health insurance status and utilization of health services in rural Northern Ghana: evidence from the introduction of the National Health Insurance Scheme

**DOI:** 10.1186/s41043-017-0128-7

**Published:** 2017-12-13

**Authors:** Philip Ayizem Dalinjong, Paul Welaga, James Akazili, Anthony Kwarteng, Martin Bangha, Abraham Oduro, Osman Sankoh, Jane Goudge

**Affiliations:** 1 Navrongo Health Research Centre, Ghana Health Service, Navrongo, Ghana; 2 Kintampo Health Research Centre, Ghana Health Service, Kintampo, Ghana; 3 INDEPTH Network Secretariat, Accra, Ghana; 4 Centre for Health Policy, School of Public Health, Faculty of Health Sciences, University of the Witwatersrand, Johannesburg, South Africa

**Keywords:** National Health Insurance, Social health insurance, Health service, Utilization, Outpatients, Inpatients, Insured, Uninsured, Ghana

## Abstract

**Background:**

Many households in low- and middle-income countries face financial hardships due to payments for health care, while others are pushed into poverty. Risk pooling and prepayment mechanisms help to lessen the impact of the costs of care as well as assisting to achieve universal health coverage (UHC). Ghana implemented the National Health Insurance Scheme (NHIS) for the promotion of access to health services for all Ghanaians. In this paper, we examined the association between health insurance status and utilization of outpatient and inpatient health services in rural poor communities.

**Methods:**

The study was a cross-sectional household survey conducted in the Kassena-Nankana districts of Northern Ghana. We conducted interviews in 11,175 households and collected data on 55,992 household members. Multiple logistic regression models were used to identify factors associated with the utilization of outpatient and inpatient health services. The dependent variables were the utilization of outpatient and inpatient health services. We adjusted for several potential socio-demographic factors associated with utilization and health insurance status.

**Results:**

Significantly, the insured had 2.51 (95% CI 2.3–2.8) and 2.78 (95% CI 2.2–3.6) increased odds of utilizing outpatient and inpatient health services respectively. Respondents with a history of recent illness or injury [32.4 (95% CI 29.4–35.8) and 5.72 (95% CI 4.6–7.1)] and poor or very poor self-reported health status [2.08 (95% CI 1.7–2.5) and 2.52 (95% CI 1.9–3.4)] and those on chronic medication [2.79 (95% CI 2.2–3.5) and 3.48 (95% CI 2.5–4.8)] also had increased odds of utilizing both outpatient and inpatient health services respectively. Among the insured, the poorest use the Community-based Health Planning and Services (CHPS) compounds, while the least poor use private clinics and public hospitals for outpatient health services. The uninsured predominately use pharmacies or licensed chemical shops (LCSs). For inpatient health services, the insured largely use public hospitals, with the uninsured using private clinics or public health centres.

**Conclusion:**

The findings suggest that being insured with the NHIS is associated with increased utilization of outpatient and inpatient health services in the study area. Overall, the NHIS can be an effective tool for achieving UHC and hence pragmatic efforts should be made to sustain it.

## Background

Access to quality health services is problematic for most people in the world. The World Health Organization (WHO) reports that about 400 million people globally, do not have access to basic quality health services and that 6% of people living in low- and middle-income countries experience extreme poverty as a result of payments for health services [1]. Universal health coverage (UHC), defined as access to basic quality health services without financial hardship, can assist in alleviating poverty. The concept is firmly rooted in the 1948 WHO Constitution; which declared health as a fundamental human right [2]. Given the importance of UHC, it has been made one of the targets for the sustainable development goals (goal 3) initiated by the United Nations in September, 2015. The target calls on all countries to endeavor to achieve UHC by year 2030. As a result, many countries including low and middle ones are pursuing various health sector reforms to pave the way for attaining UHC. These are done through the introduction of risk pooling and prepayment programs. In Africa for instance, Ghana, Rwanda, Nigeria, and Kenya have introduced health insurance schemes to allow their populations to have access to basic quality health services when needed.

### The National Health Insurance Scheme in Ghana

Ghana established a National Health Insurance Scheme (NHIS) through an Act of Parliament (Act 650) and it began full operations in 2005 [3]. The core objective of the NHIS is the provision of quality health services for all Ghanaians without any out-of pocket (OOP) payments at the point of care [4, 5]. The NHIS is functional in all metropolitan, municipal, and districts in Ghana. It is mandatory for all Ghanaians to register with the NHIS, to be able to have access to health services when needed, although this is hardly enforced. Financing sources of the NHIS include contributions from informal sector workers, the National Health Insurance levy, (a 2.5% top up on the value added tax), 2.5% deduction from the contributions of formal sector workers to the Social Security and National Insurance Trust (SSNIT), and returns on investment from the National Health Insurance Fund [4]. The minimum and maximum contributions from the informal sector to the NHIS range between GH¢7.20 and 47.70 ($8–$53) [4]. However, vulnerable groups such as pregnant women, pensioners, children below the age of 18 years, and adults above the age of 70 are all exempted from premium payment [4].

Despite such policies, studies on health insurance programs in Africa have demonstrated that the poor are not covered [6]. In Ghana for instance, evidence showed that the NHIS is not pro-poor [7–11], despite the fact that the NHIS was established as part of a poverty reduction strategy and subsequently provided exemptions for the poor [12].

Poverty has been shown to be a profound barrier to registration with the NHIS in the three regions of Northern Ghana (Upper West region, Upper East region, and Northern region) which are considered as rural and poor, compared to the rest of the country [10, 13–15]. There is limited knowledge on the effect of the NHIS on utilization of health services from rural poor communities, particularly whether health insurance enables higher utilization. Thus, this study examined the utilization of both outpatient and inpatient health services in a rural poor setting after a decade of the implementation of the NHIS. The findings will assist to understand the progress made so far in the move towards UHC, particularly the extent to which the poor have access to health services. The results are also essential for policy makers in their formulation of interventions for the poor and vulnerable.

## Methods

### Study area and design

The study was a cross-sectional household survey conducted in the Kassena-Nankana districts (East and West) of the Upper East region of Ghana. The study area has a continuous population registration system known as the Navrongo Health and Demographic Surveillance System (NHDSS). The NHDSS monitors the demographic events of the entire population of about 160,000 in 30,000 households. Data on demographic events such as births, deaths, migrations, marriages, and pregnancies are collected and updated every 3 to 4 months. Agriculture and related activities are the mainstay of the economy of the districts, with about 78.9% of the population engaged in the sector for the year 2008 [16]. Due to the dependence on agriculture and declining agricultural yields, poverty is endemic in the area [17]. Health facilities in the study area include a district hospital, 6 health centres, 27 Community-based Health Planning and Services (CHPS) compounds, 2 mission-based health centres, and 3 private clinics. The CHPS compounds are community-based health centres that are meant to provide basic primary health services for individuals in rural and remote communities. At the time of the study, the only hospital in the two districts was located in the Kassena-Nankana East district, with a 140-bed capacity. The main health centre (currently being upgraded to a district hospital) in the Kassena-Nankana West district had a 40-bed capacity. Doctor to patient ratio for the two districts was 1:31,927 in 2007 [16]. Malaria has consistently been the number one cause of outpatients’ attendance. Other top health problems include respiratory tract infections and skin and diarrheal diseases [16]. The study was conducted from July to December 2012, using a structured questionnaire.

### Sampling

In all, 12,000 households were sampled from the total number of households in the NHDSS database using a simple random sampling technique. The sampling was done using STATA 12.0 [18]. Each sampled household was visited by a fieldworker who interviewed the household head or any adult member of the household who had information on the health insurance status of the household members and their health-seeking behavior and payments for health services. We collected data on 55,992 household members from 11,175 households in the study area.

### Study variables

The dependent variables were the utilization of outpatient or inpatient health services. Outpatient utilization of service was defined as the use of any health service or purchase of drugs by an individual in the household in the last 1 month, ending on the day of interview for the study. The hospitalization of any member of the household in the last 1 year was considered as inpatient utilization, which included the day the interview was conducted. The independent variables included the socio-economic and demographic characteristics of the respondents such as wealth status, age, gender, educational status, religious affiliation, ethnicity, health insurance, geographical location, and household size. Household members with valid health insurance cards at the time of the interview were considered as being insured with the NHIS. Socio-economic status was computed from large and small household items using principal component analysis and accordingly categorized as poorest, poorer, poor, less poor, and the least poor.

### Data analysis and management

Data was entered in FoxPro 6.0 and analysed in STATA 12.0 [18]. Multiple logistic regression models were used to identify factors associated with the utilization of outpatient and inpatient health services. The inclusion of variables in the regression model was based on their level of significance (*p* < 0.05) in the bivariate analysis as well as on scientific plausibility. Reference categories were defined as those usually associated with the least utilization of health services in the literature.

## Results

### Socio-demographic characteristics of respondents

The response rate for the study was 93%. About 63% of household members were insured and 37% uninsured. Respondents who were most likely to be insured were those who were sick in the last month, as well as those with poor or very poor self-reported health status (Table 1). Women were also more likely to be insured, as were young children (0–4), those living in urban areas, the least poor, and those with tertiary education.

**Table 1:** Background characteristics of study respondents by health insurance status

Variable		Total	Insured	Uninsured
	Categories	Number(%)	Number(%)	Number(%)
Age	0–4	5952 (10.6)	4410 (74.1)	1542 (25.9)
	5–17	17,765 (31.7)	11,299 (63.6)	6466 (36.4)
	18–34	13,957 (24.9)	8960 (64.2)	4997 (35.8)
	35–59	12,352 (22.1)	7275 (58.9)	4077 (41.1)
	60–69	3467 (6.2)	1983 (57.2)	1484 (42.8)
	70+ (aged)	2499 (4.5)	1422 (56.9)	1077 (43.1)
Gender	Male	27,078 (48.4)	16,355 (60.4)	10,723 (39.6)
	Female	28,914 (51.6)	18,996 (65.7)	9918 (34.3)
Educational level	No formal education	13,519 (24.1)	7354 (54.4)	6165 (45.6)
	Primary	17,901 (32.0)	10,490 (58.6)	7411 (41.4)
	Secondary	11,580 (20.7)	8118 (70.1)	3462 (29.9)
	Tertiary	1659 (3.0)	1425 (85.9)	234 (14.1)
	Below school going age^a^	11,333 (20.2)	7967 (70.3)	3366 (29.7)
Religion	Traditional & other	22,904 (44.6)	11,864 (51.8)	11,040 (48.2)
	Christianity	27,737 (49.6)	19,471 (70.2)	8266 (29.8)
	Islam	3260 (5.8)	2579 (79.1)	681 (20.9)
Ethnicity	Kasem	28,099 (50.2)	20,091 (71.5)	8008 (28.5)
	Nankam	23,393 (41.8)	11,977 (51.2)	11,416 (48.8)
	Buli	1221 (2.2)	832 (68.1)	389 (31.9)
	Other	3279 (5.8)	1024 (86.2)	164 (13.8)
Location	Rural	48,562 (86.7)	28,992 (59.7)	19,570 (40.3)
	Urban	7430 (13.3)	6360 (85.6)	1070 (14.4)
Household size	1 member	1267 (2.3)	780 (61.6)	487 (38.4)
	2–4 members	12,392 (22.1)	7980 (64.4)	4412 (35.6)
	5+ members	42,333 (75.6)	26,585 (62.8)	15,748 (37.2)
Socio-economic status	Poorest	15,211 (27.1)	7588 (53.6)	6568 (46.4)
	Poorer	12,357 (22.1)	6182 (54.7)	5120 (45.3)
	Poor	10,454 (18.7)	6063 (58.0)	4758 (42.0)
	Less poor	10,821 (19.3)	7813 (72.2)	3008 (27.8)
	Least poor	7149 (12.8)	6270 (87.7)	879 (12.3)
Recent illness or injury	Yes	2837 (5.1)	1770 (62.4)	1067 (37.6)
	No	53,155 (94.9)	33,594 (63.2)	19,561 (36.8)
Self-assessed health status	Poor or very poor	1471 (2.6)	756 (51.4)	715 (48.6)
	Good or very good	53,827 (96.1)	34,234 (63.6)	19,593 (36.4)
	Don’t know	694 (1.3)	376 (54.2)	318 (45.8)
	Overall total	55,992 (100)	35,275 (63%)	20,717 (37%)

^a^Representing infants who have not yet attained school going age in order to be in school

### Utilization of outpatient and inpatient health services

The insured made more outpatient as well as inpatient visits compared to the uninsured (Table 2). The same trend was observed for the utilization of inpatient health services. Equally, respondents with a history of recent illness/injury, those with poor or very poor self-assessed health status as well as those on chronic medication had more visits for both outpatient and inpatient health services. However, no difference was seen in the use of health services for the various socio-economic groupings.

**Table 2:** Utilization rates for outpatient and inpatient health services

Variable		Visits		Utilization rate/per 1000	
	Categories	Outpatient	Inpatient	Outpatient	Inpatient
Health insurance status	Insured	3633	959	1.0	0.3
	Uninsured	1364	101	0.7	0.1
Age	0–4	1033	194	1.7	0.3
	5–17	1104	180	0.6	0.1
	18–34	796	244	0.1	0.2
	35–59	1270	302	1.0	0.2
	60–69	457	78	1.3	0.2
	70+ (aged)	337	62	1.4	0.3
Gender	Male	2080	442	0.8	0.2
	Female	2917	618	1.0	0.2
Educational level	No formal education	1403	228	1.0	0.2
	Primary	1186	199	0.7	0.1
	Secondary	864	256	0.8	0.2
	Tertiary	133	57	0.8	0.3
	Below school going age^a^	1411	320	1.3	0.3
Religion	Traditional & other	2070	295	0.9	0.1
	Christianity	2478	659	0.9	0.2
	Islam	241	62	0.7	0.2
Ethnicity	Kasem	2344	581	0.8	0.2
	Nankam	2128	349	.0–9.	0.2
	Buli	219	35	1.8	0.3
	Other	98	51	0.8	0.4
Location	Rural	4244	747	0.9	0.2
	Urban	753	313	1.0	0.4
Household size	1 member	159	43	1.3	0.3
	2–4 members	1256	305	1.0	0.3
	5+ members	3582	712	0.9	0.2
Socio-economic status	Poorest	1300	189	0.9	0.1
	Poorer	972	145	0.8	0.1
	Poor	867	149	0.8	0.1
	Less poor	944	262	0.9	0.2
	Least poor	704	271	0.9	0.4
Recent illness or injury	Yes	2821	776	7.7	0–9
	No	2176	243	0.5	0.2
Self-assessed health status	Poor or very poor	571	128	3.9	0.9
	Good or very good	4371	886	0.8	0.2
	Don’t know	55	5	0.8	0.1
Chronic medication	Yes	396	113	5.3	1.5
	No	4591	903	0.8	0.2
	Don’t Know	10	3	0.8	0.3

^a^Representing infants who have not yet attained school going age in order to be in school

### Factors associated with utilization of outpatient and inpatient health services

Significantly, the insured had 2.51 (95% CI 2.3–2.8) and 2.78 (95% CI 2.2–3.6) increased odds of utilizing both outpatient and inpatient health services respectively, when compared to the uninsured (Tables 3 and 4). Further, respondents with a history of recent illness or injury and poor or very poor self-assessed health status and those on chronic medication had increased odds of utilizing both outpatient and inpatient health services. But poverty was not seen to be a significant factor in the utilization of outpatient health services (Table 3), except for inpatient health services. For instance, the least poor had 2.10 (95% CI 1.5–2.9) increased odds of using inpatient health services compared to the poorest.

**Table 3:** Factors associated with utilization of outpatient health services

Variable		Outpatient health services		
	Categories	% Utilized	Unadjusted OR (95% CI)	Adjusted OR (95% CI)
Health insurance status	Insured	7.9	2.06 (1.9–2.3)	2.51 (2.3–2.8)^b^
	Uninsured	3.9	1	1
Age	0–4	14.3	4.11(3.7–4.6)	2.72 (2.2–3.3)^b^
	5–17	4.5	1.17 (1.0–1.3)	1.19 (1.0–1.4)
	18–34	3.9	1	1
	35–59	6.9	1.85 (1.7–2.1)	1.55 (1.4–1.8)^b^
	60–69	8.9	2.39 (2.1–2.8)	1.75 (1.4–2.1)^b^
	70+ (aged)	9.8	2.67 (2.3–3.1)	1.67 (1.3–2.1)^b^
Gender	Male	5.6	1	1
	Female	7.2	1.31 (1.2–1.4)	1.21 (1.1–1.3)^b^
Educational level	No education	7.2	1.29 (1.0–1.6)	1.01 (0.8–1.3)
	Primary	4.4	0.77 (0.6–0.9)	0.93 (0.7–1.2)
	Secondary	5.7	0.91 (0.7–1.1)	1.12 (0.9–1.5)
	Tertiary	10.2	1	1
	Below school going age^a^	10.2	1.88 (1.5–2.3)	1.2 (0.9–1.7)
Religion	Traditional & other	6.4	1.10 (0.94–1.29)	NS
	Christianity	6.5	1.10 (0.9–1.3)	NS
	Islam	5.9	1	NS
Ethnicity	Kasem	5.9	1	1
	Nankam	6.5	1.09 (1.0–1.2)	1.33 (1.2–1.5)^b^
	Buli	12.5	2.24 (1.9–2.7)	2.91 (2.4–3.6)^b^
	Other	7.5	1.27 (1.0–16)	0.98 (0.8–1.3)
Location	Rural	6.2	1	1
	Urban	7.9	1.28 (1.2–1.4)	1.29 (1.1–1.5)^b^
Household size	1 member	10.7	1.83 (1.5–2.2)	1.36 (1.1–1.8)^b^
	2–4 members	7.2	1.19 (1.1–1.3)	1.36 (1.1–1.8)^b^
	5+ members	6.1	1	1
Socio-economic status	Poorest	6.3	1	1
	Poorer	6.1	0.96 (0.9–1.1)	0.97 (0.9–1.1)
	Poor	6.2	0.98 (0.9–1.1)	1.10 (0.9–1.2)
	Less poor	6.3	1.00 (0.9–1.1)	1.04 (0.9–1.2)
	Least poor	7.7	1.24 (1.1–1.4)	1.23 (1.0–1.4)
Recent illness or injury	Yes	42.9	34.14 (31.3–37.2)	32.4 (29.4–35.8)^b^
	No	3.8	1	1
Self-assessed health status	Poor or very poor	25.8	5.49 (6.5–8.9)	2.08 (1.7–2.5)^b^
	Good or very good	5.9	1	1
	Don’t know	5.2	0.87 (0.62–1.2)	0.89 (0.6–1.3)
Chronic medication	Yes	32.9	7.58 (6.5–8.9)	2.79 (2.2–3.5)^b^
	No	93.9	1	1
	Don’t Know	7.4	1.2 (0.6–2.4)	0.82 (0.3–1.9)

The reference group for the calculation of the odds ratio (OR) is indicated by one

*NS* not significant in both bivariate and multivariate analyses

^a^Representing infants who have not yet attained school going age in order to be in school

^b^Significant in multivariate analyses

**Table 4:** Factors associated with utilization of inpatient health services

Variable		Inpatient health services		
	Categories	% Utilized	Unadjusted OR (95% CI)	Adjusted OR (95% CI)
Health insurance	Insured	1.4	3.49 (2.8–4.4)	2.78 (2.2–3.6)^b^
	Uninsured	0.4	1	1
Age	0–4	1.7	1.98 (1.5–2.6)	1.27 (0.9–1.9)
	5–17	0.5	0.57 (0.4–0.7)	0.66 (0.5–0.9)
	18–34	0.9	1	1
	35–59	1.4	1.63 (1.3–2.0)	1.53 (1.2–1.9)^b^
	60–69	1.4	1.65 (1.2–2.3)	1.41 (0.9–2.1)
	70+ (aged)	1.6	1.88 (1.3–2.7)	1.65 (1.1–2.5)^b^
Gender	Male	0.9	1	1
	Female	1.2	1.3 (1.1–1.6)	1.10 (0.9–1.3)
Educational level	No education	1.1	0.61 (0.4–0.9)	1.03 (0.7–1.6)
	Primary	0.5	0.29 (0.2–0.4)	0.84 (0.5–1.3)
	Secondary	1.2	0.66 (0.4–0.9)	1.25 (0.8–1.9)
	Tertiary	1.9	1	1
	Below school going age^a^	1.4	0.73 (0.5–1.1)	1.47 (0.9–2.5)
Religion	Traditional & other	0.7	0.49 (0.4–0.7)	NS
	Christianity	1.3	0.87 (0.6–1.2)	NS
	Islam	1.4	1	NS
Ethnicity	Kasem	1.2	1	1
	Nankam	0.8	0.66 (0.6–0.8)	0.95 (0.8–1.2)
	Buli	1.6	1.41 (0.9–2.2)	1.71 (1.1–2.7)^b^
	Other	2.4	2.12 (1.4–3.1)	1.17 (0.8–1.8)
Location	Rural	0.9	1	1
	Urban	2.2	2.62 (2.2–3.1)	1.32 (1.0–1.7)
Household size	1 member	2.4	2.7 (1.9–3.9)	1.55 (1.0–1.5)
	2–4 members	1.4	1.62 (1.4–1.9)	1.27 (1.0–1.5)
	5+ members	0.9	1	1
Socio-economic status	Poorest	0.7	1	1
	Poorer	0.8	1.14 (0.9–1.5)	1.10 (0.8–1.5)
	Poor	0.8	1.12 (0.8–1.5)	1.10 (0.8–1.5)
	Less poor	1.2	1.73 (1.3–2.3)	1.48 (1.1–1.9)^b^
	Least poor	2.3	3.40 (2.6–4.4)	2.10 (1.5–2.9)^b^
Recent illness or injury	Yes	0.8	8.23 (6.9–9.9)	5.72 (4.6–7.1)^b^
	No	6.0	1	1
Self-assessed health status	Poor or very poor	4.9	5.43 (4.2–6.9)	2.52 (1.9–3.4)^b^
	Good or very good	0.9	1	1
	Don’t know	0.7	0.77 (0.32–1.9)	0.81 (0.3–2.0)
Chronic medication	Yes	8.8	10.27 (7.9–13.4)	3.48 (2.5–4.8)^b^
	No	0.9	1	1
	Don’t Know	1.7	1.79 (0.4–7.2)	1.19 (0.3–5.0)

The reference group for the calculation of the odds ratio (OR) is indicated by one

*NS* not significant in both bivariate and multivariate analyses

^a^Representing infants who have not yet attained school going age in order to be in school

^b^Significant in multivariate analyses

### Health insurance status and types of health providers visited

We examined the different types of health providers utilized by respondents for both outpatient and inpatient health services (Table 5). Among the insured, there was some variation as to which facilities were utilized for outpatient health services, with the poorest using CHPS compounds. The least poor used private clinics and public hospitals instead, for their outpatient health needs. But the uninsured predominately use pharmacies or licensed chemical shops (LCSs). These facilities are solely responsible for the sale of drugs to clients. For inpatient health services, the insured largely use public hospitals, although utilization by the least poor is more than double that of the poorest. Within the uninsured, utilization of inpatient health service is much lower, with the poorest not using public hospitals at all, but rather private clinics or public health centres.

**Table 5:** Health insurance status and types of health care providers visited

		Outpatient rate (per 1000)			Inpatient rate (per 1000)		
	Provider	Insured	Uninsured	Total	Insured	Uninsured	Total
Population	CHPS compounds (public)	44.6	5.4	30.1	1.0	0.2	0.7
	Pharmacies or LCSs	14.9	38.3	23.6	0.0	0.0	0.0
	Public health centres	13.0	1.9	8.9	1.2	0.9	1.1
	Private clinics	10.4	2.1	7.3	0.4	0.1	0.3
	Public hospitals	19.3	1.8	12.8	23.4	3.0	15.9
	Traditional healers	2.8	1.8	2.4	0.1	0.2	0.1
Poorest	CHPS compounds (public)	56.3	6.9	33.3	0.5	0.0	0.3
	Pharmacies or LCSs	12.2	35.6	23.4	0.0	0.0	0.0
	Public health centres	20.2	2.8	12.1	2.0	1.8	1.9
	Private clinics	6.1	1.5	4.0	0.3	2.2	1.2
	Public hospitals	15.0	1.9	8.9	18.1	0.0	9.7
	Traditional healers	2.7	2.4	2.6	0.1	0.0	0.1
Least poor	CHPS compounds (public)	20.3	1.1	17.9	1.4	0.0	1.2
	Pharmacies or LCSs	18.6	26.9	19.6	0.0	0.0	0.0
	Public health centres	5.8	0.0	5.0	0.3	0.0	0.3
	Private clinics	20.0	4.3	18.0	1.1	1.1	1.1
	Public hospitals	35.0	3.2	31.0	38.3	3.2	33.8
	Traditional healers	3.1	3.2	3.1	0.0	0.0	0.0

## Discussion

The study examined the association between the insurance status of individuals and the utilization of outpatient and inpatient health services. It also examined household members’ utilization of various types of health providers. Being insured was significantly associated with higher odds of utilizing both outpatient and inpatient health services. The finding is consistent with other studies on utilization in other parts of Ghana and Africa [19, 20]. A study in Accra, Ghana’s capital city, showed that registered clients of the NHIS were significantly more likely to receive prescriptions, visit clinics, and use formal health facilities when in need [21]. Another study among households in the Mfantseman municipality (central region) in Ghana reported that insured members were more likely to utilize outpatient health services compared to the uninsured [22]. Studies in Rwanda have also shown an increase in the utilization of health services under the community-based health insurance [23–26]. Thus, the results from our study conducted in a rural poor setting do not differ from findings from other similar settings. A recent report from the Ministry of Health showed a continued rise in the utilization of health services under the NHIS. From the period 2003 to 2009, outpatient attendance per capita rose from 0.50 to 0.81 [27]. The observed increased in the utilization of health services could be linked to the introduction of the NHIS. However, these findings could have financial implications for the sustainability of the NHIS, given that those more likely to need care are registered and subsequently utilizing health services, rather than all Ghanaians being registered and contributing financially.

The findings also suggest that the existence of the NHIS has led to increased use of the CHPS compounds by the poorest who were insured, as reported in other studies [28–30] Thus, distance as a barrier to the utilization of health services has been reduced with the introduction of the CHPS compounds. A recent report by the Ministry of Health indicated that CHPS compounds in Ghana were responsible for about 5% of the total outpatient attendance in 2011 [28]. We recommend that more CHPS compounds be built to help bring primary health services to the doorsteps of Ghanaians and therefore help strengthen the gatekeeper system and promote more broadly UHC.

Uninsured respondents, regardless of their socio-economic standing, were utilizing outpatient health services from pharmacies or licensed chemical shops, requiring direct OOP payments with its attendant financial implications. We recommend a rigorous effort on the part of the NHIS to register more individuals in the NHIS in order to reduce OOP payments and to promote utilization. The findings show that socio-economic standing did not pose a barrier to outpatient health service use; rather, it influenced the type of health provider used. The study has helped to shed light on the type of health provider utilized by the insured and uninsured.

### Limitations of the study

One of the limitations of this study was that household heads were responsible for answering all the questions for their household members. In the absence of the household head, an adult member of the household with information about the household members responded for the household members. It may be problematic for the household head or the representative to provide accurate responses for all household members, especially on their health status as well as their health-seeking behavior. However, considering that the household sizes are generally small (about five), and being a rural area where information is often shared among household members, we do not expect so much variation in the responses from individuals from the same household.

As the study was cross-sectional, we are unable to determine changes in utilization across time. The sampled insured and uninsured households may systematically be different from each other due to unobserved factors such as some aspect of wealth or income, perceived benefits of insurance, and perceived health status which might influence registration with the NHIS and subsequent utilization of health services. Thus our findings may be biased by these unobservable factors. Nonetheless, the study showed a strong association between having an insurance cover and the utilization of health services. The implementation of the NHIS in Ghana provides important lessons for other low- and middle-income countries that are planning or have implemented prepayment programs in one way or the other.

## Conclusion

Being insured with the NHIS is associated with increased utilization of both outpatient and inpatient health services compared to the uninsured. As expected, respondents considered to be in need of health services (those with poor or very poor self-reported health status, history of recent illness or injury, and those on chronic medication) were found to have increased odds of utilizing health services under the NHIS. The insured were using outpatient health services from CHPS compounds and that of inpatient health services from public hospitals. However, the uninsured utilized outpatient health services from pharmacies or licensed chemical shops. The NHIS can be an effective tool for achieving UHC and hence pragmatic efforts should be made to sustain it.

## Abbreviations

CHPS: Community-based Health Planning and Services
CI: Confidence interval
LCSs: Licensed chemical shops
NHDSS: Navrongo Health and Demographic Surveillance System
NHIS: National Health Insurance Scheme
OOP: Out-of pocket
OR: Odds ratio
UHC: Universal health coverage
WHO: World Health Organization
